# Stable incidence and continued improvement in short term mortality of *Staphylococcus aureus* bacteraemia between 1995 and 2008

**DOI:** 10.1186/1471-2334-12-260

**Published:** 2012-10-17

**Authors:** Niels Mejer, Henrik Westh, Henrik C Schønheyder, Allan G Jensen, Anders R Larsen, Robert Skov, Thomas Benfield

**Affiliations:** 1 Department of Infectious Diseases, Hvidovre University Hospital, Hvidovre, Denmark; 2 Faculty of Health Sciences, University of Copenhagen, Copenhagen, Denmark; 3 Department of Clinical Microbiology, Hvidovre University Hospital, Hvidovre, Denmark; 4 Department of Clinical Microbiology, Aarhus University Hospital, Aalborg, Denmark; 5 Staphylococcal Laboratory, Statens Serum Institut, Copenhagen, Denmark; 6 Pfizer, Ballerup, Denmark; 7 Clinical Research Centre, Hvidovre University Hospital, Hvidovre, Denmark

**Keywords:** Bacteraemia, Epidemiology, Incidence, Mortality, Comorbidity, Alcoholism, *Staphylococcus aureus*, Charlson comorbidity index

## Abstract

**Background:**

The objective of this study was to assess temporal changes in incidence and short term mortality of *Staphylococcus aureus* bacteraemia (SAB) from 1995 through 2008.

**Methods:**

The study was conducted as a nation-wide observational cohort study with matched population controls. The setting was hospitalized patients in Denmark 1995-2008. Uni- and multivariate analyses were used to analyze the hazard of death within 30 days from SAB.

**Results:**

A total of 16 330 cases of SAB were identified: 57% were hospital-associated (HA), 31% were community-acquired (CA) and 13% were of undetermined acquisition. The overall adjusted incidence rate remained stable at 23 per 100 000 population but the proportion of SAB cases older than 75 years increased significantly. Comorbidity in the cohort as measured by Charlson comorbidity index (CCI) score and alcohol-related diagnoses increased over the study period. In contrast, among the population controls the CCI remained stable and alcohol-related diagnoses increased slightly. For HA SAB crude 30-day mortality decreased from 27.8% to 21.8% (22% reduction) whereas the change for CA SAB was small (26.5% to 25.8%). By multivariate Cox regression, age, female sex, time period, CCI score and alcohol-related diagnoses were associated with increased mortality regardless of mode of acquisition.

**Conclusions:**

Throughout a 14-year period the overall incidence of SAB remained stable while the overall short term prognosis continued to improve despite increased age and accumulation of comorbidity in the cohort. However, age and comorbidity were strong prognostic indicators for short term mortality.

## Background

During the last decades general improvements in health and socioeconomic conditions in many industrialized countries have led to an older population [1]. In Denmark, individuals older than 65 years increased only modestly from 16% to 18% between 1990 and 2010 but the proportion of individuals older than 90 increased by 72% [2]. Age is one of the strongest determinants of risk of infectious disease [3]. Older age is further associated with an increased risk and prevalence of comorbidity [4]. The combination of an ageing population and associated comorbidity may lead to an increase in the incidence of sepsis and bloodstream infection. This was evident throughout the 1980’s and 1990’s for *Staphylococcus aureus* bacteraemia (SAB) and bacteraemia in general as shown by us and others [5-7]. During the same time frame, the prevalence of several other risk factors has increased. These include increased use of cancer chemotherapy, immunosuppressive therapy, intravascular devices, and invasive medical and surgical procedures. Taken together ageing, accumulation of comorbidity and other risk factors may influence both the incidence and prognosis of SAB.

Although much attention has been drawn towards methicillin-resistant *S. aureus* (MRSA) in recent years, methicillin-susceptible *S. aureus* (MSSA) continues to constitute the most common type of SAB in most parts of the world. In Denmark MRSA SAB is infrequent. Using a nationwide cohort we investigated contemporary changes in the incidence rate (IR) of SAB (> 99% MSSA), temporal changes in age and underlying morbidity and assessed the effect of each on short term outcome from MSSA SAB from 1995 to 2008.

## Methods

### Study population

The register-based matched cohort was created by merging data from four national registries based on the unique civil registration number assigned to each Danish citizen [8]. The study was approved by the Ethics Committee for the Municipalities of Copenhagen and Frederiksberg (01–369 ⁄ 93) and the Danish Data Protection Agency (2009-41-4179).

The Danish Staphylococcal Bacteraemia Study Group has carried out a continuous, nationwide registration of SAB in Denmark since 1956 [6,9]. The present study defined a case as an individual with a first episode of SAB identified via the registry between January 1, 1995 and December 31, 2008. Discharge records for each admission with SAB were reviewed [6,9]. We defined hospital-associated SAB (HA SAB) as an infection acquired after 48 hours of hospitalisation, an infection in a patient in ambulatory care in a specialized hospital department, or a case residing in a nursing home. Community-acquired SAB (CA SAB) was defined as an infection present at the time of hospitalization in patients not meeting the definition of HA SAB. Methicillin resistance was determined by antimicrobial susceptibility testing and confirmed by the presence of the *mec*A gene [10,11].

Information on the number of persons alive in Denmark on January 1 from 1995 through 2008 was obtained from Statistics Denmark (Danmarks Statistik at http://www.dst.dk). These included total number and subgroups defined by age and gender.

The Danish Civil Registration System which is updated daily, tracks changes in vital status and migration for the entire Danish population [8]. For each case, we randomly selected 10 control subjects, matched by age (year of birth) and sex. We used the risk set sampling technique (i.e. eligible control subjects had to be alive and at risk of a first hospitalization with SAB on the date the corresponding case was admitted [12]).

Data on comorbidity was collected from the National Patient Registry. This registry is updated monthly and holds information on all hospital admissions and discharge diagnoses of all patients treated in a Danish hospital since January 1, 1977 as well as all outpatient contacts since January 1, 1995. The International Classification of Diseases (ICD)-8 was used from 1977 through 1993; and ICD-10 from 1994. Comorbidity prior to the SAB admission was assessed by estimating the Charlson comorbidity index (CCI) score [13,14]. Briefly, the CCI is a validated score system used to asses patients’ comorbid conditions in longitudinal studies [15], and takes into account both the number and severity of comorbid disease. We calculated the CCI score for cases and controls by gathering diagnoses from the National Patient Registry from up to 10 years prior to the SAB index date and excluding diagnoses from the admission with SAB. To avoid the influence of re-registration of diagnoses, each of the 17 Charlson diagnostic categories could only contribute one time to the overall index. We defined three levels of comorbidity for categorizing patients in this study: none (patients without underlying disease), intermediate (weighted index of comorbidity CCI 1–2) and high (CCI ≥ 3). Alcohol related comorbidity is not included in the CCI score and was assessed separately as previously described [16,17]. We considered the following diagnosis codes: for ICD-10 classification: F10, G31.2, G62.1, G72.1, I42.6, K29.2, K86.0, K70, R78.0, T51, Z72.1, and for ICD-8 classification: 291 (Alcoholic psychosis), 303 (Alcoholism), 979 (Alcohol in combination with specified medicinal agents), 980 (Toxic effect of alcohol), 577.10 (Chronic pancreatitis due to alcohol), 571.09 (Cirrhosis of the liver due to alcohol), 571.10 (Steatosis of the liver due to alcohol). ICD-10 K70 (cirrhosis) is included in the CCI score but was only assessed for alcohol-related conditions.

### Statistics

Annual data were divided into five periods (1995–1997, 1998–2000, 2001–2003, 2004–2006 and 2007–2008) for the assessment of temporal changes, and age was divided into 6 groups (<1, 1–15, 16–35, 36–55, 56–75, >75). Using the cumulative incidence proportion method to calculate incidence rates, that assumes that all individuals of a given birth cohort remain alive for a given year, indicated that incidence rates increased over time. However, annual survival rates for individuals older than 65 improved significantly during the study, leading to an average increase in days at risk of SAB in the older age strata in more recent time periods. E.g. among population controls >75 years the 30-day- and 1-year mortality proportion decreased from 7,0% and 51,4% in 1995–1997 to 1,2% and 12,8% in 2007–2008, respectively. Therefore, we adjusted the number of days at risk in each gender- and age stratum by a correction factor derived from annual survival among the population controls. Incidence rates with 95% CIs were calculated based on these adjusted data. Categorical variables were compared by chi-square test. Ninety days survival curves were constructed by the Kaplan-Meier method, and the proportional hazards assumption was evaluated visually on survival plots. Mortality rate ratios with 95% CIs were calculated using Cox proportional hazards models. Unvariate and multivariate Cox regression was performed to determine the mortality rate ratios within 30 days of SAB. All variables in the multivariate analysis were analysed using the forced entry procedure. Analyses were performed using PASW v.18 (SPSS Inc., Chicago, IL, USA).

## Results

During the study period, 17 450 individuals were hospitalized with a first episode of SAB. A total of 820 of these were excluded due to a temporary or invalid personal identifier (n=652), negative follow up time (n=142), no sample date (n=18) or a duplicated person identifier (n=8). MRSA was rare (<1%, Table 1).

**Table 1:** **Characteristics of cases with hospital-associated and community-acquired *Staphylococcus aureus *bacteraemia in Denmark from 1995-2008**

	**Hospital-associated SAB N = 9412 (56.6%)**	**Community-acquired SAB N = 5074 (30.5%)**
**MRSA**		
No	9332(99.2%)	5039(99.3%)
Yes	80(0.8%)	35(0.7%)
**Age, years**		
<1	337(3.6%)	58(1.1%)
1-15	232(2.5%)	235(4.6%)
16-35	535(5.7%)	405(8.0%)
36-55	1733(18.4%)	938(18.5%)
56-75	3982(42.3%)	1734(34.2%)
>75	2593(27.5%)	1704(33.6%)
**Sex**		
Female	3755(39.9%)	1927(38.0%)
Male	5657(60.1%)	3147(62.0%)
**CCI score**		
0	2822(30.0%)	2293(45.2%)
1-2	3868(41.1%)	1801(35.5%)
> 2	2722(28.9%)	980(19.3%)
**Alcohol-related disorders**		
No	8585(91.2%)	4532(89.3%)
Yes	827(8.8%)	542(10.7%)
**Year**		
1995-1997	1893(20.1%)	989(19.5%)
1998-2000	1992(21.2%)	1261(24.9%)
2001-2003	2010(21.4%)	893(17.6%)
2004-2006	2171(23.1%)	1155(22.8%)
2007-2008	1346(14.3%)	776(15.3%)

SAB: *Staphylococcus aureus* bacteraemia; MRSA: Methicillin-resistant *Staphylococcus aureus*.

CCI: Charlson comorbidity index.

### Characteristics of cases and controls

Fifty-seven percent of cases were HA and 31% were CA. In 13% of cases the place of acquisition could not be determined. The characteristics of HA and CA cases are shown in Table 1. Overall, patients were in their mid-sixties and the majority were male.

The proportion of cases older than 75 years increased from 23.2% in 1995–1997 to 29.8% in 2007–2008 (p = 0.0001), and the proportion of cases aged 1–55 years decreased during the same period from 37.1% to 28.1% (p = 0.0001). The proportion of cases aged <1 years and 56–75 years were stable.

The proportion of cases who had a CCI score > 0 increased during the study period from 60.8% in 1995–1997 to 69.5% in 2007–2008 (p = 0.0001), of which the proportion of individuals with a CCI score > 2 increased from 19% to 32% (p = 0.0001). The proportion of cases with a CCI score increased with age although fewer cases older than 75 years compared to the 56–75 year old stratum had a CCI score greater than 0 (72.0% vs. 74.8%, p = 0.001). Patients with HA SAB had higher CCI scores than CA SAB. Among HA SAB 70,0% had a CCI greater than 0 compared to 54,8% among CA SAB (p = 0.0001).

The proportion of cases with pre-admission alcohol-related diagnoses increased from 7% to 12% between 1995–1997 and 2007–2008 (p = 0.0001). Alcohol-related diagnoses were more frequent among CA SAB compared to HA SAB (p = 0.0001).

The distribution of CCI (p = 0.0001) and alcohol-related disorders (p = 0.0001) among the age- and sex matched controls differed from cases. For controls 23% had a CCI score of 1–2 and only 6.0% had a CCI score > 2. Over time the proportion of controls with a CCI score > 0 decreased from 31.4% in 1995–1997 to 28.0% in 2007–2008 (p = 0.0001), while the proportion of controls with pre-admission alcohol-related diagnoses increased from 1.5% to 2.1% (p = 0.0001).

### Incidence rates of SAB

The adjusted overall IR (22.7/100 000 population (95%CI: 22.4-23.1/100 000)) of first time SAB remained constant from 1995 to 2008 (Table 2). IRs increased with age and were higher among males (27.9/100.000 population (95%CI: 27.4-28.5/100 000)) than females (17.6/100.000 population (95%CI: 17.2-18.1/100.000)). The IRs decreased slightly from 1995 to 2008 among persons aged 1–75 years and were stable among cases aged <1 and >75 years (Table 2).

**Table 2:** Incidence rates* of *Staphylococcus aureus *bacteraemia in Denmark from 1995-2008

**Year**	**1995-1997**	**1998-2000**	**2001-2003**	**2004-2006**	**2007-2008**
**All**	23.4(22.6-24.2)	23.5(22.8-24.3)	21.2(20.5-21.9)	23.4(22.6-24.1)	21.8(20.9-22.7)
**Sex**					
Male	28.3(27.1-29.5)	29.4(28.2-30.6)	26.1(25.0-27.2)	28.6(27.5-29.8)	27.1(25.8-28.6)
Female	18.7(17.8-19.7)	17.9(17.0-18.8)	16.5(15.6-17.4)	18.2(17.3-19.1)	16.6(15.5-17.7)
**Age, years**					
<1	40.5(31.8-49.2)	37.1(28.7-45.6)	42.3(33.2-51.4)	57.0(46.3-67.6)	52.6(40.1-65.1)
1-15	5.3(4.4-6.1)	4.6(3.8-5.4)	3.1(2.5-3.7)	3.4(2.8-4.1)	3.5(2.7-4.4)
16-35	7.3(6.5-8.1)	6.9(6.1-7.6)	4.3(3.6-4.9)	4.3(3.7-4.9)	4.0(3.2-4.7)
36-55	16.8(15.6-18.1)	15.7(14.5-16.8)	13.4(12.3-14.4)	13.7(12.6-14.8)	13.5(12.2-14.8)
56-75	58.9(55.8-62.0)	51.6(48.9-54.3)	45.3(42.9-47.7)	46.7(44.3-49.0)	43.5(40.8-46.2)
> 75	114.2(106.3-122.0)	120.7(113.4-128.1)	112.7(105.9-119.5)	126.7(119.6-133.8)	107.7(99.7-115.6)

* Adjusted incidence rates per 100 000 population years, (): 95% Confidence interval.

### Mortality

The overall cumulative mortality among cases within 30 days from SAB was 25.7%. The median survival of HA, CA and undetermined acquisition SAB was 556 days (501–610), 836 days (721–951) and 476 days (386–566), respectively.

**Figure 1:**
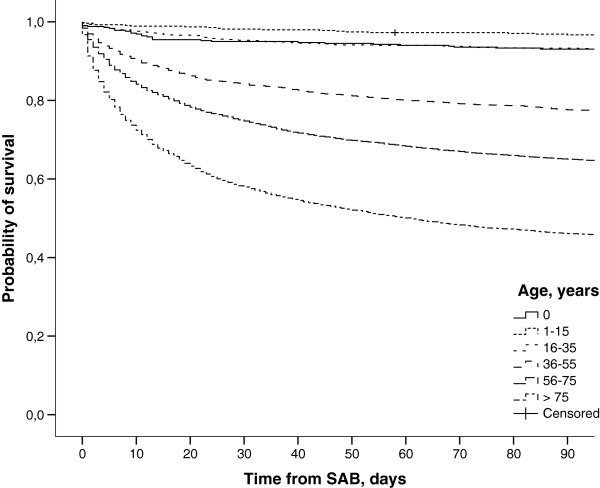
**Survival curves of 16630 cases with *Staphylococcus aureus *bacteraemia between 1995 and 2008 according to age.**

**Figure 2:**
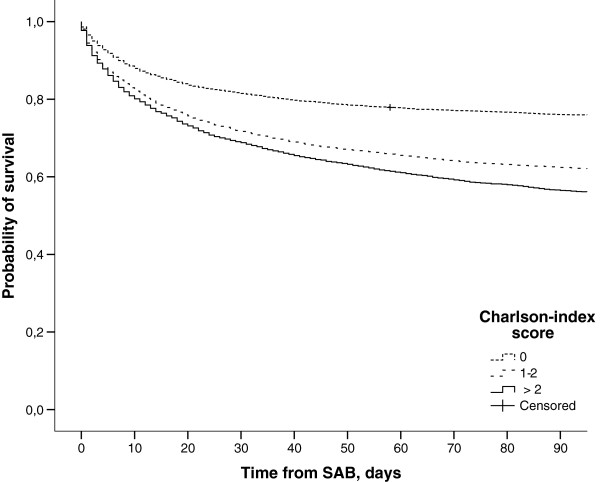
**Survival curves of 16630 cases with *Staphylococcus aureus *bacteraemia between 1995 and 2008 according to Charlson comorbidity index score.**

There was a significant impact of origin, age, gender, CCI, alcohol-related disorders and time period on mortality (Tables 3 and 4). Due to few deaths among SAB patients <36 years the periodic 30 day mortality proportions in these age groups were not presented. The highest impact on mortality was noted with age (Figure 1). The impact diminished in multivariate analyses, but remained the most influential. Second and third highest impact was seen with alcohol-related disorders and comorbidity assessed by CCI (Figure 2). By multivariate analyses the impact of alcohol-related disorders increased after adjustment for age whereas the impact of CCI decreased. This was seen among both HA and CA SAB. Multivariate analyses showed improved survival over time particularly among cases of HA but also CA bacteraemia. Gender had the lowest impact on mortality and men had lower mortality in the adjusted analysis.

**Table 3:** **Thirty-day mortality proportions from *Staphylococcus aureus *bacteraemia in Denmark between 1995 and 2008**

**Year**	**1995-1997**	**1998-2000**	**2001-2003**	**2004-2006**	**2007-2008**
**All**	0.27(0.25-0.28)	0.26(0.25-0.28)	0.26(0.24-0.27)	0.26(0.25-0.27)	0.23(0.21-0.25)
**Sex**					
Female	0.29(0.26-0.31)	0.27(0.25-0.30)	0.28(0.26-0.30)	0.28(0.25-0.30)	0.25(0.22-0.28)
Male	0.26(0.24-0.28)	0.25(0.24-0.27)	0.24(0.22-0.26)	0.25(0.23-0.27)	0.22(0.20-0.24)
**Age, years**					
36-55	0.19(0.17-0.22)	0.17(0.14-0.19)	0.15(0.12-0.18)	0.15(0.12-0.17)	0.11(0.08-0.14)
56-75	0.29(0.27-0.32)	0.26(0.24-0.28)	0.23(0.21-0.25)	0.24(0.22-0.26)	0.23(0.21-0.26)
> 75	0.45(0.42-0.48)	0.44(0.41-0.47)	0.42(0.39-0.45)	0.42(0.39-0.45)	0.37(0.33-0.40)
**CCI**					
0	0.20(0.18-0.22)	0.20(0.18-0.23)	0.18(0.15-0.20)	0.16(0.14-0.18)	0.17(0.15-0.20)
1-2	0.30(0.28-0.32)	0.29(0.27-0.31)	0.29(0.26-0.31)	0.27(0.25-0.30)	0.25(0.22-0.28)
> 2	0.34(0.31-0.38)	0.30(0.27-0.33)	0.30(0.27-0.33)	0.34(0.31-0.36)	0.26(0.23-0.29)
**Alcohol-related disorders**	0.34(0.28-0.40)	0.32(0.27-0.37)	0.31(0.26-0.35)	0.32(0.27-0.37)	0.30(0.24-0.35)

CCI: Charlson comorbidity index, (): 95% Confidence interval.

**Table 4:** **Multivariate analysis of factors associated with mortality among cases of hospital-associated and community-acquired *Staphylococcus aureus *bacteraemia in Denmark from 1995-2008**

	**Hospital-associated SAB**		**Community-acquired SAB**	
	**30-d mortality (%)**	**Adjusted MRR (95% CI)**	**30-d mortality (%)**	**Adjusted MRR (95% CI)**
**Overall**	2368/9412 (25.2%)		1439/5074 (28.4%)	
**Age, years**				
1 >	19/337 (5.6%)	0.45 (0.28-0.73)	1/58 (1.7%)	0.13 (0.02-0.93)
1-15	9/232 (3.9%)	0.27 (0.14-0.53)	1/235 (0.4%)	0.03 (0.04-0.23)
16-35	32/535 (6.0%)	0.39 (0.27-0.56)	15/405 (3.7%)	0.25 (0.14-0.42)
36-55	282/1733 (16.3%)	1.0	152/938 (16.2%)	1.0
56-75	983/3982 (24.7%)	1.7 (1.5-1.9)	483/1734 (27.9%)	1.8 (1.5-2.2)
> 75	1043/2593 (40.2%)	3.2 (2.8-3.7)	787/1704 (46.2%)	3.6 (3.0-4.4)
**Sex**				
Female	988/3755 (26.3%)	1.0	616/1927 (32.0%)	1.0
Male	1380/5657 (24.4%)	0.90 (0.83-0.98)	823/3147 (26.2%)	0.82 (0.74-0.92)
**CCI score**				
0	527/2822 (18.7%)	1.0	435/2293 (19.0%)	1.0
1-2	1049/3868 (27.1%)	1.3 (1.1-1.4)	606/1801 (33.6%)	1.3 (1.1-1.5)
> 2	792/2722 (29.1%)	1.4 (1.2-1.6)	398/980 (40.6%)	1.6 (1.4-1.9)
**Alcohol-related disorders**				
No	2113/8585 (24.6%)	1.0	1251/4532 (27.6%)	1.0
Yes	255/827 (30.8%)	1.7(1.5-2.0)	188/542 (34.7%)	1.9(1.6-2.2)
**Year**				
1995-1997	526/1893 (27.8%)	1.0	262/989 (26.5%)	1.0
1998-2000	502/1992 (25.2%)	0.83 (0.74-0.94)	365/1261 (28.9%)	1.00 (0.85-1.17)
2001-2003	512/2010 (25.5%)	0.79 (0.70-0.89)	257/893 (28.8%)	0.89 (0.75-1.06)
2004-2006	535/2171 (24.6%)	0.74 (0.66-0.84)	355/1155 (30.7%)	0.90 (0.76-1.05)
2007-2008	293/1346 (21.8%)	0.65 (0.56-0.75)	200/776 (25.8%)	0.74 (0.61-0.89)

SAB: *Staphylococcus aureus* bacteraemia; MRR: Mortality rate ratio; CI: confidence interval, CCI: Charlson comorbidity index.

## Discussion

In a cohort of more than 16 000 individual episodes of SAB, we show that the incidence of first time SAB remained stable across age groups, origin and degree of comorbidity from 1995 to 2008 after several decades of increasing incidence rates. Further, we document that overall short term survival rates continued to improve although mortality rates were higher with increasing age and level of comorbidity.

The stable incidence of SAB in the observation period was also found elsewhere [18], but is somewhat surprising. A continued increase in incidence was expected given an increasing larger proportion of elderly with more comorbidity and in a time with more chemotherapy, immunosuppressive treatment and increased use of invasive procedures and devices. A possible explanation may be that although the proportion of elderly is expanding, the elderly are ageing healthier than just a decade ago. Our analysis of the control population certainly showed a considerable improvement in survival over time in support of this.

This study identified high risk of SAB among persons <1 year. This finding is consistent with other contemporary studies [19-21] and warrants further studies on SAB among neonates.

The short term mortality associated with SAB decreased by 14% from 1995–1997 to 2007–2008 in spite of the presence of more comorbidity and increased age. In fact, the total number of deaths attributed to SAB declined for the first time since 2000 because the incidence rate also remained stable. We speculate that the improved gains may have been accomplished through healthier ageing and improved treatment of comorbid diseases, but many factors may be at play. Still, further improvement in the treatment of SAB must be accomplished if the improvement in the prognoses of SAB is to follow the improvement seen in the age-matched general population.

The strengths of this study lies in its size, the longitudinal and population-based design, the low frequency of MRSA which enabled a clean focus on MSSA, the uniform registration system, and the inclusion of randomly selected age- and sex matched population controls that allowed for adjustment of a baseline mortality risk. There are also potential limitations. First, the discharge data, which the assessment of comorbidity was built upon, may have contained errors, but this misclassification bias would be non-differential between cases and controls. Second, the temporal rise in CCI and alcohol-related diagnoses among cases could have been caused by an intensified registration of diagnoses. This is, however, unlikely since diagnoses included in the CCI score among population controls remained constant. Third, using CCI as a aggregated estimate of comorbidity has limitations: the CCI unlikely included all comorbid diseases and the improved prognosis of many diseases including cancer and HIV/AIDS in the study period may have overadjusted the impact of CCI on mortality late in the observation period compared to early. Fourth, surveillance bias among the increasing proportion of cases with pre-SAB comorbidity could explain part of the improved prognosis of SAB.

Fifth, a potential major limitation of the study, is its classification of HA and CA SAB. The present study included community onset healthcare-related SAB as HA SAB which could make comparison to data from the recent literature difficult.

## Conclusion

In conclusion, the overall IR of first time SAB did not increase from 1995 to 2008 as would be expected from the changing epidemiological profile of the Danish population and the increased medical activity. Further, the short term prognosis of SAB improved; especially among HA SAB. Although the over-all risk of dying from SAB declined, the short term mortality of SAB is still high, especially among the old and comorbid, which continues to constitute a growing proportion of the Danish SAB patients. This study underscores the importance of adjustment for age and comorbidity when comparing risk of and mortality from SAB sequentially or between countries. In addition, it highlights the potential benefits of future studies in prevention and treatment of SAB among the old and comorbid patients.

## Abbreviations

SAB: *Staphylococcus aureus* bacteraemia; MRSA: Methicillin-resistant *Staphylococcus aureus*; MSSA: Methicillin-susceptible *Staphylococcus aureus*; CCI: Charlson comorbidity index; HA: Hospital-associated; CA: Community-acquired; IR: Incidence rate; CI: Confidence interval.

## Competing interests

A.G. Jensen has been working at Pfizer Denmark since 2005 but Pfizer Denmark has no financial interests in- or support to the present study. All other authors declare that they have no competing interests.

## Authors’ contributions

TB conceived the study idea. NM and TB designed the study. NM, HW, HCS, AGJ, ARL, RS and TB collected the data and NM analyzed the data. NM and TB interpreted the findings. NM wrote the initial draft. All authors edited the manuscript and approved the final version. TB is the guarantor.

## Pre-publication history

The pre-publication history for this paper can be accessed here:

http://www.biomedcentral.com/1471-2334/12/260/prepub
